# Comparison of Liver Recovery After Sleeve Gastrectomy and Roux-en-Y-Gastric Bypass

**DOI:** 10.1007/s11695-021-05390-1

**Published:** 2021-04-04

**Authors:** Sophia M.-T. Schmitz, Andreas Kroh, Alexander Koch, Jonathan F. Brozat, Christine Stier, Ulf P. Neumann, Tom F. Ulmer, Patrick H. Alizai

**Affiliations:** 1 Department of General-, Visceral- and Transplantation Surgery, RWTH Aachen University Hospital, Pauwelsstr. 30, 52074 Aachen, Germany; 2 Department of Gastroenterology, Digestive Diseases and Intensive Care Medicine, RWTH Aachen University Hospital, Pauwelsstr. 30, 52074 Aachen, Germany; 3 Obesity Center NRW, Sana Kliniken, Hürth, Germany; 4 Department of Surgery, Maastricht University Medical Center, P. Debyelaan 25, 6229 HX Maastricht, The Netherlands

**Keywords:** NAFLD, Liver function, LiMAx, Sleeve gastrectomy, Roux-en-Y-gastric bypass, Bariatric surgery

## Abstract

**Background:**

Nonalcoholic fatty liver disease (NAFLD) is a common condition in patients with obesity. Bariatric surgery has often been proposed as a viable treatment option, but the ideal surgical procedure remains unclear. Inconsistently, reports on postoperative deterioration of liver function put further doubt on which technique to apply. Aim of this study was to assess the impact of Roux-en-Y-gastric bypass (RYGB) and sleeve gastrectomy (SG) on the postoperative recovery of liver function.

**Methods:**

A total of 175 patients with obesity that underwent bariatric surgery in our institution were included in this prospective cohort study. BMI, laboratory values, and liver function capacity (using LiMAx) were assessed preoperatively and at 6 and 12 months postoperatively. Generalized linear model (GLM) was performed to determine variables influencing liver function capacity after the operation.

**Results:**

Prior to operations, 64% of patients presented with a diminished liver function capacity, as measured by LiMAx test. Liver function capacity significantly recovered after 12 months in the SG group (300 μg/kg/h preop vs. 367 μg/kg/h postop) but not in the RYGB group (306 μg/kg/h preop vs. 349 μg/kg/h). Preoperative factors impeding liver function recovery included type 2 diabetes mellitus (T2DM), weight, male sex, AST/thrombocyte ratio (APRI), and gamma-glutamyltransferase (GGT).

**Conclusion:**

Bariatric surgery, especially sleeve gastrectomy, leads to an improvement of liver function. However, in some patients with T2DM, higher preoperative weight and male sex postoperative deterioration of liver function capacity may occur.

**Graphical abstract:**

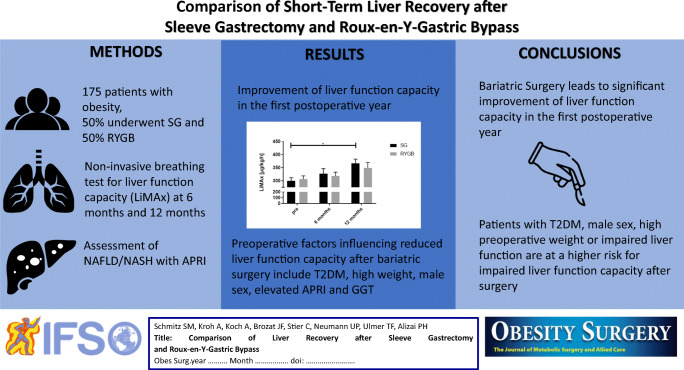

## Introduction

Nonalcoholic fatty liver disease (NAFLD) is a progressive condition affecting a large number of patients worldwide, with an estimated prevalence of 25% [1]. Obesity is found to be an underlying condition for NAFLD frequently and can be considered as the hepatic manifestation of the metabolic syndrome [2]. The rising incidence of NAFLD is closely linked to the rising incidence of obesity [1, 3]. Prevalence of NAFLD in patients undergoing bariatric surgery has been described in up to 95% of cases [4]. Weight loss improves NAFLD and reduces its likelihood of progression to nonalcoholic steatohepatitis (NASH) and cirrhosis [5–10]. The positive impact of bariatric surgery on altitude of liver enzyme elevation has been assessed in a large prospective intervention study [11]. Histologically, grade of NAFLD ameliorates after bariatric surgery, as measured by NAFLD activity score (NAS) [6, 9, 12–14]. Even liver fibrosis can be resolved after bariatric surgery in some cases [6, 9, 14–17]. However, there are also reports of acute liver failure or worsening of preexisting liver injury following bariatric surgery [6, 18, 19].

As of today, the right surgical procedure as a treatment option for patients with NAFLD is subject to intense discussions. Some studies suggest sleeve gastrectomy (SG) and Roux-en-Y-gastric bypass (RYGB) to be of equal effectiveness in amelioration of NAFLD [10, 12, 13], while others favor either RYGB [6, 17, 20] or SG [5, 16, 21]. Thus, the aim of this study was to compare postoperative recovery of liver function between RYGB and SG and to assess risk factors for potential deterioration of liver function capacity.

## Material and Methods

### Study Design

The present study was conducted between 2013 and 2019 as a prospective cohort study at RWTH Aachen University Hospital.

Indication for surgery was defined through an interdisciplinary board as follows: body mass indices (BMI) of > 40 kg/m^2^ or > 35 kg/m^2^ with weight-related comorbidities. Patients with a BMI < 50 kg/m^2^ were submitted to intensive preoperative weight loss therapy prior to operation. Patients with < 15% reduction in body weight were then transferred to surgery. Operative procedure (sleeve gastrectomy or Roux-en-Y-gastric bypass) was determined according to individual conditions (comorbidities and previous abdominal operations) and patients’ preferences. In patients with a history of diabetes or esophagogastric reflux disease, Roux-en-Y-gastric bypass (RYGB) was preferred over sleeve gastrectomy (SG). In some cases with high abdominal fat masses, SG had to be performed due to anatomical causes. RYGB was performed with an antecolic Roux-en-Y-configuration with a biliopancreatic limb of 50–75cm (proximal RYGB). Patients under 18 years of age or with a history of alcohol consumption (> 20g/day) were excluded. Diagnosis of diabetes was made on the basis of HbA1C levels over 6.5%, fasting plasma glucose > 126 mg/dl, or oral glucose tolerance test 140 to 199 mg/dl. Informed consent was obtained from all individual participants included in the study. All patients receive specific dietetic recommendations after surgery. Recommendations include balanced meals with small portions and a diet low in calories, sweets, and fat with a high protein intake. After both procedures, a multivitamin supplementation is recommended and after RYGB, an additional calcium supplementation. Further supplementations are recommended according to blood test results. Medication for diabetes of hypertension is reduced or discontinued as soon as possible. Participants of this study underwent the non-invasive LiMAx liver function capacity test and blood collection prior to operation, as well as 6 and 12 months after surgery. All data, including clinical data such as age, weight, and height, were stored in a secured, pseudonymized database. Percent excess BMI loss (%EBMIL) was calculated according to Deitel et al. [22].

All procedures performed in studies involving human participants were in accordance with the ethical standards of the institutional and/or national research committee and with the 1964 Helsinki declaration and its later amendments or comparable ethical standards. Ethical approval was granted by our local Ethics Committee.

### Laboratory Tests

Blood samples were drawn after overnight fasting the day before the operation. Biochemical parameters were determined at the Institute of Clinical Chemistry of our University Hospital. Normal ranges of alanine aminotransferase (ALT) and aspartate aminotransferase (AST) are < 50 U/L; for gamma-glutamyltransferase (GGT) < 40 U/L. Normal range of platelet (PLT) was defined as 150–400/nL, while overall percentage of glycated hemoglobulin (HbA1c) was considered non-pathological between 4 and 6%. APRI was calculated by dividing AST through platelets [23, 24].

### Liver Function Capacity

LiMAx test was used for non-invasive measurement of liver function capacity. It is based on metabolism of ^13^C-methacetin by the hepatic cytochrome P450 1A2 system (CYP1A2). The test is performed via intravenous injection of 2 mg/kg body weight ^13^C-methacetin (Euriso-top, Saint-Aubin Cedex, France). Metabolization of ^13^C-methacetin into acetaminophen and ^13^CO_2_ is measured by examination of exhaled ^13^CO_2_. Metabolization is measured through real-time point-of-care (POC) breath sampling with a laser-based nondispersive isotope-selective infrared spectroscope (FLIP2, Humedics, Berlin, Germany). The set up allows for analysis of functional capacity of the CYP1A2 system (hereafter referred to as liver functioning capacity). A physiological liver function capacity is indicated by values of > 315 μg/kg/h.

### Statistical Analysis

Statistical analysis was performed using Graph Pad Prism® v7, IBM SPSS® v25, and Microsoft Excel®. Outliers with a value higher or lower than two times the standard deviation (SD) of the mean were excluded from further analyses. Values are presented as mean and standard error of the mean (SEM) or median and interquartile range (IQR) unless stated otherwise. One-way ANOVA and the two-sided *t*-test were used to compare means. Comparison of surgical procedure and impairment of liver function capacity was done with chi-square test. Generalized linear model (GLM) was used for multivariate analysis. The β coefficients in the GLM were used to assess the effect of the independent variables. Higher coefficients resemble higher association. A *p* < 0.05 was considered statistically significant.

## Results

### Patient Characteristics

A total of 175 patients were included in this study; 50% of patients (*n* = 88) underwent SG and 50% (*n* = 87) underwent RYGB. Mean BMI prior to operation was 52.7 kg/m^2^ (SEM 0.64) and 73% of patients (*n* = 129) were female. There was no statistically significant difference in BMI prior to operation for patients that underwent SG when compared to patients that underwent RYGB (*p* = 0.87). Patients in the SG group were more likely to be male (37% vs. 15% in the RYGB group) and suffer from hypertension (63% vs. 51%) and type 2 diabetes mellitus (T2DM) (38% vs. 24%). For further details on the study population, see Table 1. Overall mean BMI decreased significantly after 6 months (39.7 kg/m^2^, SEM 0.56) and 12 months (35.7 kg/m^2^, SEM 0.55); 12 month postoperative %EMBIL was higher after RYGB when compared to SG (66%, SEM 2.1 vs. 57%, SEM 2.4; *p* = 0.013) (see Table 2).

**Table 1:** Study population prior to operation

	Total	SG	RYBG	*p*-value
*n*	175	88	87	
Female	129 (73%)	55 (63%)	74 (85%)	0.003*
BMI (kg/m^2^)	52.7 (0.64)	53.5 (1.01)	51.8 (0.78)	0.087
LiMAx (μg/kg/h)	302.8 (8.9)	300.1 (12.0)	305.6 (13.2)	0.756
Abnormal liver function test results (LiMAx < 315 μg/kg/h)	103 (64%)	51 (62%)	52 (65%)	0.807
APRI	0.099 (0.003)	0.1 (0.005)	0.097 (0.004)	0.328
T2DM	54 (31%)	33 (38%)	21 (24%)	0.036*
Arterial hypertension	99 (66%)	55 (63%)	44 (51%)	0.046*

**Table 2:** Study population 6 and 12 months after surgery

		Total	SG	RYGB	*p*-value
BMI (kg/m^2^)	6 months	39.7 (0.56)	41.1 (0.95)	38.5 (0.62)	0.446
	12 months	35.7 (0.55)	37.7 (0.95)	34.2 (0.6)	0.132
%EBMIL	6 months	49 (1.3)	46 (2)	51 (1.6)	0.147
	12 months	62 (1.6)	57 (2.4)	66 (2.1)	0.013*
LiMAx (μg/kg/h)	6 months	322.6 (12.1)	324.8 (19.4)	320.6 (14.9)	0.862
	12 months	359.6 (12.6)	367.0 (15.7)	348.6 (21)	0.485
Abnormal liver function test results (LiMAx < 315 μg/kg/h)	6 months	43 (47.3%)	21 (47.7%)	22 (46.8)	0.930
	12 months	48 (64%)	31 (68.9%)	17 (56.7%)	0.280
APRI	6 months	0.0831 (0.002)	0.0838 (0.004)	0.0824 (0.003)	0.143
	12 months	0.0837 (0.003)	0.0774 (0.004)	0.0892 (0.004)	0.103
T2DM	6 months	17 (12%)	9 (13%)	8 (11%)	0.704
	12 months	16 (11%)	8 (11%)	8 (11%)	0.884

### Preoperative Liver Function

Prior to surgery, mean LiMAx value was 303 μg/kg/h (SEM 8.9). A LiMAx value prior to operation could be obtained from 162 patients, *n* = 80 patients in the RYGB group and *n* = 82 in the SG group. There was no difference between the SG and the RYGB groups (*p* = 0.807); 103 patients (64%) had a score below the normal value of 315 μg/kg/h. Mean APRI prior to operation in all patients was 0.099 (SEM 0.003) with no statistically significant difference between SG and RYGB groups (*p* = 0.328) (see Table 1). APRI could be obtained from 164 patients prior to operation.

### Postoperative Liver Function

**Fig. 1:**
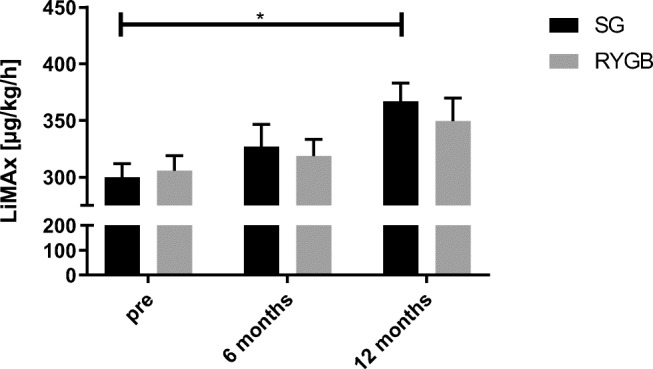
Liver function capacity (LiMAx) in patients prior to surgery and after 6 and 12 months. Abbreviations: SG, sleeve gastrectomy; RYGB, Roux-en-Y-gastric bypass; * statistically significant using one-way ANOVA, *p* = 0.021

A LiMAx test could be obtained from 91 patients after 6 months (47 patients in the RYGB group and 44 patients in the SG group) and 75 patients after 12 months (30 patients in the RYGB group and 45 patients in the SG group). After 1 year, LiMAx values improved significantly only in the SG group (300 μg/kg/h preop vs. 367 μg/kg/h postop, *p* = 0.021), while values in the RYGB group showed no statistically significant improvement (306 μg/kg/h preop vs. 349 μg/kg/h postop, *p* = 0.45) (Fig. 1). A weak but significant inverse correlation between %EBMIL and percentual change of liver function capacity could be observed at 6 months (*Y* = − 0.70 × X + 48.23, *R*^2^ = 0.10, *p* = 0.002), but not at 12 months following bariatric surgery (*Y* = 0.11 × X + 23.76, *R*^2^ = 0.002, *p* = .714). Six months after surgery, 47% of patients showed an impaired liver function capacity, while 12 months after surgery, 64% of patients had an impaired liver function capacity. Chi-square test revealed no difference between patients after SG and RYGB (*p* = 0.930 and 0.280, respectively).

**Fig. 2:**
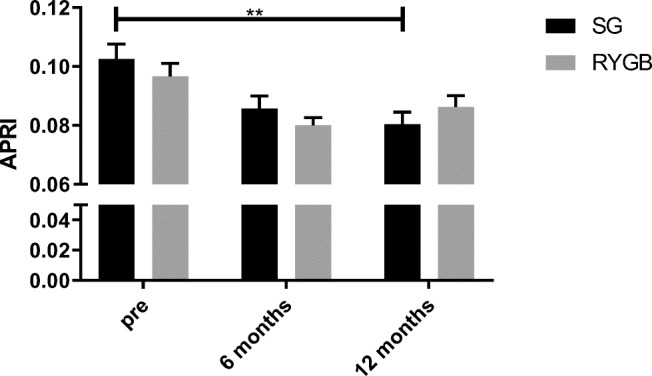
APRI in patients prior to surgery and after 6 and 12 months. Abbreviations: SG, sleeve gastrectomy; RYGB, Roux-en-Y-gastric bypass; ** statistically significant using one-way ANOVA, *p* = 0.007

APRI could be calculated in 142 patients after 6 months (76 patients in the RYGB group and 66 patients in the SG group) and 122 patients after 12 months (65 patients in the RYGB group and 57 patients in the SG group). APRI index showed a similar pattern as LiMAx with improvement after 12 months in the SG group (0.1 preop vs. 0.081 postop, *p* = 0.007) but not in the RYGB group (0.097 preop vs. 0.086 postop, *p* = 0.67) (Fig. 2).

### Factors Influencing Liver Function Capacity

Generalized linear model was performed for liver function capacity measured by LiMAx at 6 and 12 months postoperatively. Covariates included preoperative LiMAx, age, APRI, BMI, and body weight, as well as laboratory values HbA1c, AST, ALT, and GGT. Fixed factors included were sex, T2DM, and hypertension.

*At 6 months after surgery*, T2DM was associated with a 71-unit decrease in LiMAx in patients after RYGB (*β* = −70.7, SE 33.4, *p* = 0.034) but not after SG (*β* = 2.37, SE 59.2, *p* = 0.97). For patients undergoing RYGB, each kilogram of weight prior to operation was associated with a 2-unit decrease in LiMAx (*β* = −2, SE 0.9, *p* = 0.027). Of all laboratory values taken into account, only GGT revealed a significant association to liver function capacity after 6 months (*β* = −1.1, SE 0.5, *p* = 0.03). For patients undergoing SG, only preoperative GGT revealed a significant association with LiMAx after 6 months with an increase of 2.2 units for each unit of GGT (*β* = 2.2, SE 0.8, *p* = 0.006). For a summary of all variables, see Table 3.

**Table 3:** Multivariate analysis of factors influencing liver function capacity 6 months after surgery

Operative procedure	Independent factors	Estimate, β	SE	95% Confidence interval	*p*-value
RYGB	Intercept	285.3	172.4	− 52.5–623.27	0.098
	Sex	6.2	42.9	− 77.8–90.2	0.885
	T2DM	− 70.7	33.4	5.2–136.1	0.034*
	Arterial hypertension	− 10.8	26.8	− 63.3–41.8	0.688
	Age	0.6	1.1	− 1.6–2.8	0.597
	Weight	− 1.9	0.9	− 3.8 to − 0.22	0.027*
	BMI	1.0	2.7	− 4.2–6.2	0.710
	LiMAx	0.5	0.1	0.3–0.7	0.000*
	APRI	660.9	269.0	133.6–1188.2	0.014*
	AST	1.6	2.1	− 2.6–5.8	0.462
	ALT	− 1.7	1.1	− 3.8–0.5	0.137
	GGT	− 1.1	0.5	− 2.1 to − 0.1	0.030*
	HbA1c	31.0	24.3	− 16.7–78.6	0.203
	%EBMIL 6 months after surgery	− 1.6	0.8	− 3.3–0.0	0.056
SG	Intercept	746.2	332.2	95.2–1397.2	0.025*
	Sex	128.3	70.5	− 9.9–266.6	0.069
	T2DM	− 2.4	59.2	− 113.7–118.5	0.968
	Arterial hypertension	23.4	48.0	− 70.7–117.5	0.626
	Age	− 6.1	3.1	− 12.3–0.1	0.052
	Weight	− 2.1	2.0	− 6.0–1.8	0.291
	BMI	− 1.4	4.5	− 10.2–7.5	0.763
	LiMAx	0.2	0.2	− 0.2–0.7	0.350
	APRI	− 950.5	569.0	− 2065.8–164.7	0.095
	AST	− 3.6	3.8	− 11.2–4.0	0.351
	ALT	4.3	2.6	− 0.8–9.4	0.096
	GGT	2.2	0.8	0.6–3.8	0.006*
	HbA1c	27.5	40.2	− 51.2–106.2	0.494
	%EBMIL 6 months after surgery	− 2.0	1.1	− 4.2–0.3	0.082

Abbreviations: *BMI*, body mass index; *LiMAx*, liver function capacity test LiMAx; *SG*, sleeve gastrectomy; *RYGB*, Roux-en-Y-gastric bypass; *SE*, standard error; *T2DM*, type 2 diabetes mellitus; *APRI*, aspartate aminotransferase (AST) to platelet ratio; *AST*, aspartate aminotransferase; *ALT*, alanine aminotransferase; *GGT*, gamma-glutamyl transferase; *HbA1c*, glycated hemoglobin; *%EBMIL*, percentage excess BMI loss; * statistically significant using generalized linear model

*At 12 months after surgery*, preoperative weight was associated with a decreased liver function capacity in patients after RYGB (*β* = −3.1, SE 1.5, *p* = 0.043). After SG, male gender was associated with a 140-unit decreased liver function capacity compared to female gender (*β* = −140.3, SE 51.4, *p* = 0.006). LiMAx and APRI prior to operation were both associated with an increased liver function capacity after 12 months in patients (*β* = 0.6, SE 0.1, *p* = 0.000 and *β* = 1241.8, SE 395, *p* = 0.002, respectively). For a summary of all variables after 12 months, please refer to Table 4.

**Table 4:** Multivariate analysis of factors influencing liver function capacity 12 months after surgery

Operative procedure	Independent factors	Estimate, β	SE	95% Confidence interval	*p*-value
RYGB	Intercept	184.7	428.3	− 654.6–1024.1	0.666
	Sex	93.5	60.2	− 24.6–211.6	0.121
	T2DM	− 37.2	60.6	− 156.0–81.5	0.539
	Arterial hypertension	− 45.7	60.6	− 164.5–73.1	0.450
	Age	− 3.0	2.4	− 7.8–1.7	0.211
	Weight	− 3.1	1.5	− 6.2 to − 0.1	0.043*
	BMI	7.4	5.5	− 3.4–18.2	0.182
	LiMAx	0.4	0.2	0.1–0.7	0.007*
	APRI	− 262.3	717.7	− 1669.1–1144.4	0.715
	AST	− 1.4	3.6	− 8.4–5.6	0.699
	ALT	− 0.1	2.4	− 4.8–4.5	0.950
	GGT	− 1.1	1.0	− 3.1–1.0	0.301
	HbA1c	51.3	48.1	− 43.0–145.5	0.286
	%EBMIL 12 months after surgery	0.3	3.2	− 6.0–6.6	0.935
SG	Intercept	98.1	210.5	− 314.4–510.6	0.641
	Sex	− 140.3	51.4	− 241.0 to − 39.6	0.006*
	T2DM	− 6.5	38.0	− 80.8–67.9	0.864
	Arterial hypertension	− 1.4	40.0	− 79.9–77.1	0.972
	Age	3.5	2.1	− 0.740–7.7	0.106
	Weight	1.1	1.3	− 1.5–3.7	0.406
	BMI	1.3	3.0	− 4.5–7.1	0.659
	LiMAx	0.6	0.1	0.3–0.8	0.000*
	APRI	1241.8	395.0	467.6–2016.1	0.002*
	AST	− 1.4	2.8	− 6.9–4.187	0.632
	ALT	− 3.6	2.3	− 8.1–1.0	0.123
	GGT	1.1	0.8	− 4.4–2.6	0.165
	HbA1c	− 50.2	25.4	− 99.9 to − 0.4	0.049*
	%EBMIL 12 months after surgery	0.8	1.3	− 1.7–3.3	0.514

Abbreviations: *BMI*, body mass index; *LiMAx*, liver function capacity test; *SG*, sleeve gastrectomy; *RYGB*, Roux-en-Y-gastric bypass; *SE* standard error; *T2DM* type 2 diabetes mellitus; *APRI*, aspartate aminotransferase (AST) to platelet ratio; *AST*, aspartate aminotransferase; *ALT*, alanine aminotransferase; *GGT*, gamma-glutamyl transferase; *HbA1c*, glycated hemoglobin; *%EBMIL*, percentage excess BMI loss; * statistically significant using generalized linear model

## Discussion

Bariatric surgery presents an accepted treatment modality for NAFLD [6, 11, 12, 17, 25, 26]. However, evidence on the modality of choice with regard to NAFLD (SG or RYGB) is sparse and a statement regarding the operative approach is lacking in the guidelines [26, 27]. While some studies suggest superiority for one of the operative procedures, others advocate their equality [12, 25]. A paramount concern about bariatric surgery in patients with chronic liver disease is potential worsening of preexisting liver conditions, underlining the importance of NAFLD assessment prior to surgery [19, 26, 28, 29]. Aim of this study was to assess the impact of SG and RYGB on recovery or worsening of liver function in patients with obesity after bariatric surgery.

Different non-invasive screening tools for NAFLD are used in daily clinical routine. NAFLD practice guidelines by the American Association for the Study of Liver Diseases recommend the usage of the aspartate aminotransferase (AST) to platelet ratio (APRI), among others [27]. APRI proved particularly useful for diagnosing NAFLD in patients with obesity [30]. The non-invasive LiMAx test also reached a high sensitivity and specificity in detecting NAFLD in patients with obesity and has been developed for evaluation of liver function capacity in liver surgery [30–32]. Alterations of the biliary flow as present in patients after RYGB do not seem to influence the postoperative liver function capacity measured by LiMAx test, as the test has been validated in patients undergoing major liver resection with biliary reconstruction [33].

In our study, we found significantly improved mean liver function capacities as measured by both LiMAx test and APRI in patients who underwent SG but not RYGB (see Figs. 1 and 2). Our data concurs closely with Billeter et al., who found improved liver function following SG in comparison to RYGB in patients with elevated liver enzymes and type 2 diabetes mellitus (T2DM) [21]. Although statistical significance was not reached, a prospective cohort study further described similarly improved liver regeneration in patients who underwent SG, but not RYGB [34]. Contradictory to these results of enhanced function capacity in patients undergoing sleeve gastrectomy, a recently published meta-analysis found no significant difference in NAFLD remission between RYGB and SG as correlated through alanine transaminase, aspartate transaminase, NAFLD activity score, and NAFLD fibrosis score [12].

While mean LiMAx scores improved significantly after SG and showed a positive trend after RYGB, the percentage of patients presenting with impaired liver function capacity remained similar succeeding both procedures (see Table 2). An explanation can be that while most patients’ liver function improves after surgery, other patients might suffer from impairment of liver function. Furthermore, even patients with improved liver function capacity after surgery might still suffer from impaired liver function due to severely impaired initial liver function capacity. This ultimately leads to the important question, which patients present with a constant or worsening liver function capacity after bariatric surgery.

There is growing evidence that major changes in liver function and volume occur within the first 6 months after surgery [19, 20]. In our study, generalized linear model revealed significant influences for T2DM, preoperative weight, preoperative LiMAx, APRI, and GGT on liver function capacity after 6 months in patients after RYGB (see Table 3). In contrast, 6 month liver function capacity for SG patients seemed only affected by GGT, showing a slight, albeit, positive impact. %EBMIL was not associated with a decreased liver function capacity after 12 months neither in the SG nor in the RYGB group but revealed a trend towards a decreased liver function capacity in both groups after 6 months (see Table 4). Additionally, %EBMIL revealed a negative correlation with percentage change in LiMAx value after 6 months. Taken together, these findings indicate that rapid weight loss in the first 6 months after surgery might be associated with an initial worsening of liver function. Supporting this, a recent randomized controlled trial by Kalinowski et al. found a deterioration in liver function 1 month after surgery that was resolved after 12 months [19]. In this study, patients with NAFLD were at a higher risk of postoperative impaired liver function after RYGB than patients after SG. Likewise, Nickel et al. described an increased APRI shortly (1 month) after bariatric surgery [20]. However, they also found significant improvement of APRI 12 months after surgery with no difference between SG and RYGB [20]. Correspondingly, Yeo et al. report on a positive correlation of weight loss and amelioration of NAFLD after 12 months but not after 6 months. Mean BMI prior to operation in that study was 42 kg/m^2^ and hence much lower than in ours (mean BMI 53 kg/m^2^). One factor influencing early postoperative liver function capacity in patients after bariatric surgery is the development of portomesenteric vein thrombosis [35]. This rare but severe condition appears to evolve more frequently after SG than after RYGB, most likely due to locally diminished blood flow after dissection of the greater curvature [36, 37].

Our findings furthermore suggest an influence of T2DM on regeneration of liver function after metabolic surgery. This is of high clinical importance due to the high prevalence of diabetes in patients with obesity. In patients who underwent RYGB, presence of T2DM was associated with a decrease in LiMAx at 6 months after surgery (see Table 3). For SG, patients with T2DM showed no significant decrease in LiMAx after 6 months. Our data indicate a slight advantage of SG in patients with T2DM with regard to early postoperative liver function capacity. In another matched pair study, SG has also been described superior to RYGB in terms of ameliorating liver function in patients with T2DM [21].

Other factors influencing postoperative liver function capacity include APRI, GGT, and preoperative LiMAx. In particular for RYGB, we found an association of higher GGT levels with lower postoperative liver function capacity values after 6 months. Surprisingly, in SG, GGT levels showed a positive impact on liver function capacity after 6 months (see Table 3).

These findings have some clinical implications. Liver function shows a significant improvement after SG but not after RYGB. In terms of preexisting liver diseases, SG is preferred in patients with manifest cirrhosis due to a lower complication profile by some authors [5, 29, 38–40]. Furthermore, SG has been described as being more cost-effective than RYGB in patients with NASH cirrhosis [41]. While RYGB has recently been proposed as superior in EWL for patients that suffer from super obesity [42], two-stage RYGB with an initial sleeve gastrectomy followed by RYGB yields comparable results [43]. As higher weight was associated with a decreased liver function capacity after 6 months in patients with RYGB but not SG, our data encourage choosing SG as a first step with possible step-up to RYGB after initial weight loss in patients with high preoperative weight. To sum up, in patients with high preoperative weight and underlying preexisting liver disease, SG can be considered less harmful than RYGB. Furthermore, our findings reveal risk factors for impaired liver function capacity and indicate which patients might benefit from a closer monitoring of liver function postoperatively.

There are some limitations to be mentioned. One limitation is the high number of missing data for LiMAx after 6 and 12 months which might be due to the fact that LiMAx test is not available everywhere. However, we could obtain APRI values from almost all patients and could show a similar tendency. The results were not correlated to histological findings of liver biopsies. Furthermore, shear wave elastography and magnetic resonance elastography have recently been gaining importance in the diagnosis of fibrosis in NAFLD and might be a valuable option for preoperative evaluation of liver function [44, 45]. Another limitation is the relatively small number of patients included and the lack of randomization. Notwithstanding these limitations, as guidelines for the selection of the operative procedure in NAFLD are lacking, this study provides encouraging results towards patient selection and monitoring of patients at risk for worsening of liver function after bariatric surgery.

## Conclusion

In patients undergoing bariatric surgery, liver function improved significantly 12 months after sleeve gastrectomy, but not after Roux-en-Y-gastric bypass. We found a negative correlation between weight loss and amelioration of liver function. Factors associated with a decreased liver function capacity included type 2 diabetes mellitus, high preoperative weight, impaired APRI, elevated GGT, and male sex. Therefore, patients with these constellations should receive monitoring of liver function after the operation.

Data indicated as *n* (percent) or mean (SEM). Abbreviations: *BMI*, body mass index; *LiMAx*, liver function capacity test LiMAx; *SG*, sleeve gastrectomy; *RYGB*, Roux-en-Y-gastric bypass; *SEM*, standard error of the mean; *APRI*, aspartate aminotransferase (AST) to platelet ratio; *T2DM*, type 2 diabetes mellitus; * statistically significant using chi-square test

Data indicated as *n* (percent) or mean (SEM). Abbreviations: *BMI*, body mass index; *LiMAx*, liver function capacity test LiMAx; *SG*, sleeve gastrectomy; *RYGB*, Roux-en-Y-gastric bypass; *SEM*, standard error of the mean; *APRI*, aspartate aminotransferase (AST) to platelet ratio; * statistically significant using two-sided *t*-test
